# High-Resolution X-Ray Tomography: A 3D Exploration Into the Skeletal Architecture in Mouse Models Submitted to Microgravity Constraints

**DOI:** 10.3389/fphys.2018.00181

**Published:** 2018-03-06

**Authors:** Alessandra Giuliani, Serena Mazzoni, Alessandra Ruggiu, Barbara Canciani, Ranieri Cancedda, Sara Tavella

**Affiliations:** ^1^Sezione di Biochimica, Biologia e Fisica Applicata, Dipartimento di Scienze Cliniche Specialistiche e Odontostomatologiche, Università Politecnica delle Marche, Ancona, Italy; ^2^Dipartimento di Medicina Sperimentale, Universita' di Genova and Ospedale Policlinico San Martino, Genova, Italy

**Keywords:** high-resolution tomography, bone microarchitecture, synchrotron radiation, microgravity, animal model, mice

## Abstract

Bone remodeling process consists in a slow building phase and in faster resorption with the objective to maintain a functional skeleton locomotion to counteract the Earth gravity. Thus, during spaceflights, the skeleton does not act against gravity, with a rapid decrease of bone mass and density, favoring bone fracture. Several studies approached the problem by imaging the bone architecture and density of cosmonauts returned by the different spaceflights. However, the weaknesses of the previously reported studies was two-fold: on the one hand the research suffered the small statistical sample size of almost all human spaceflight studies, on the other the results were not fully reliable, mainly due to the fact that the observed bone structures were small compared with the spatial resolution of the available imaging devices. The recent advances in high-resolution X-ray tomography have stimulated the study of weight-bearing skeletal sites by novel approaches, mainly based on the use of the mouse and its various strains as an animal model, and sometimes taking advantage of the synchrotron radiation support to approach studies of 3D bone architecture and mineralization degree mapping at different hierarchical levels. Here we report the first, to our knowledge, systematic review of the recent advances in studying the skeletal bone architecture by high-resolution X-ray tomography after submission of mice models to microgravity constrains.

## Introduction

Bone is considered one of the most complex tissues in the body because of the continuous remodeling process that it undergoes in physiological conditions. This occurs not only in order to bear mechanical loading, but also to inhibit the damage consequent to fatigue demand. Furthermore, remodeling acts in repairing fractures, in achieving the viability of the osteocytes and calcium homeostasis.

Bone cells act synergistically, increasing or decreasing bone mass based on several factors: the osteoblasts, the bone-forming cells, regulate the deposition of the bone matrix molecules, including type I collagen and a variety of other non-collagenous proteins; the osteoclasts, multinucleated giant cells, are responsible for the mineralized bone matrix resorption (Tavella et al., 2012).

Normally, the remodeling process consists in a slow building phase and faster resorption with the objective to maintain a functional skeleton locomotion to counteract the Earth gravity. Thus, when exercise and movements are reduced, as it happens in bed-rest prolonged conditions or during spaceflights, the skeleton does not act against gravity, with a rapid decrease of bone mass and density, rendering bone fragile (Zhang et al., 2008).

The bone architecture and its remodeling were traditionally studied by X-ray radiography, but this method presents several important limitations, like misleading superimposition of anatomic structures.

In the 1970s, the development of the first equipment for computed tomography (CT), capable of producing three-dimensional (3D) virtual reconstructions of objects, in a non-destructive way and with contrast discrimination up to 10^3^ times better than conventional radiographs (Claesson, 2001), revolutionized and gave new stimuli to a more in-depth study of the bone structure.

Computed microtomography (microCT) is based on the same physical and methodologic principles of conventional CT currently used for medical imaging diagnostics. While the CT systems typically have a maximum linear resolution of about 500 μm, some microCT devices reach a spatial resolution up to 0.3 μm (Cancedda et al., 2007; Rominu et al., 2014), with an increase of about three orders of magnitude.

Subsequently, allowing accurate non-destructive 3D examination of objects, the microCT was used to reconstruct the complex architecture of bone tissue at a high resolution. In the field of bone research, different methods have now been developed to extract quantitative architectural parameters from the microCT images. For instance, the 3D mean intercept length (MIL) method is able to measure the trabecular thickness and spacing based on structural geometry assumptions (Hildebrand and Ruegsegger, 1997a). It is also feasible to skip these assumptions, extracting parameters that are model independent (Hildebrand and Ruegsegger, 1997b).

In this scenario, synchrotron radiation (SR) has proved to be of critical relevance in microCT investigations because of its specific features, including the high signal-to-noise ratio, a high photon flux to achieve measurements at high spatial resolution, and the possibility to tune the beam energy, avoiding beam hardening effects.

SR-microCT successfully supported several studies of 3D bone architecture and of mineralization degree mapping at different hierarchical levels (Nuzzo et al., 2002; Bousson et al., 2004; Lane et al., 2005), including also the imaging of the lacuno-canalicular network (Langer et al., 2012; Peyrin et al., 2014). Moreover, SR-based microCT was also employed to reconstruct, at high resolution, the complex architecture of bone tissue in different genetic/environmental conditions (Martín-Badosa et al., 2003; Costa et al., 2013; Hesse et al., 2014), and it is also increasingly becoming a powerful tool for the engineered bone characterization in several areas of skeletal research (Cancedda et al., 2007; Giuliani et al., 2013, 2014; Mazzoni et al., 2017).

However, these recent advances in microCT physics/technology have further stimulated the study of weight-bearing skeletal sites by novel imaging approaches, not only in the validated acquisition of functional images but also to investigate degenerative diseases with induced site-specific bone loss, for instance resulting from weightless environment during spaceflight.

Here we report the first, to our knowledge, systematic review of the recent advances in studying the skeletal bone architecture by high-resolution tomography after submission of mice animal models to microgravity constrains.

## Applying microgravity constraints to bone evaluated by high-resolution X-Ray tomography

The growing interest in this specific research started after the Gemini, Apollo, and Skylab missions, when the astronauts experienced a strong bone demineralization coupled to an increased calcium excretion, with negative consequences on bone turnover comparable to those on subjects subjected to long bed-rest (Wronski and Morey, 1983).

Indeed, bed-rest is the most similar condition on Earth to reduced-gravity on the skeleton, with impaired ratio between bone formation and resorption, causing an accelerated bone loss by an increased osteoclast activity that has been demonstrated in different observations (Donaldson et al., 1970; Leblanc et al., 1990, 1995).

Studies on cosmonauts aboard the Russian MIR space station confirmed the previous findings with a significant bone loss in the weight-bearing tibia and unaltered bone mass in the non-weight-bearing radius (Vico et al., 2000).

Analogous analysis of crewmembers after 4- to 6-month flights on the International Space Station (ISS) provided morphometric data on cortical and trabecular bone in spine and hip sites using quantitative computed tomography (QCT). It was observed that there was no compartment-specific loss of bone in the spine and that the cortical bone mineral loss in the hip was mainly due to endocortical thinning (Lang et al., 2004).

However, the weaknesses of the previously reported studies was two-fold: on the one hand the research suffered the small sample size of almost all human spaceflight studies, on the other the results were affected by the partial volume averaging on cortical bone measurements, due to the fact that the observed structures were small compared with the spatial resolution of the imaging device (Lang et al., 2004).

These reasons have led to the growing use of animal models, ranging from rats (Cosmi et al., 2009; Keune et al., 2015) to fish (Chatani et al., 2015), to increase the sample size by providing more significant statistical data, and to increasingly perform more informative microCT investigations, being a high-resolution 3D imaging technique able to study smaller bone structures.

### Mice in space

Most studies on microgravity have been carried out using the mouse and its various strains. These experiments have been conducted in space aboard the Space Shuttles and, for each mission, ever more sophisticated animal habitation cages have been developed. Indeed, environmental conditions are expected to affect physiology and behavior of mice both on Earth and in Space. Blottner (Blottner et al., 2009) analyzed the effects of cage confinement on the weight-bearing musculoskeletal system of 24 wild-type C57BL/6JRj mice housed for 25 days in the MIS (Mice In Space) habitat prototype, which was ground-based and fully automated. The system was a portion of the MSRM1 (Mouse Science Reference Module) device produced by Alcatel Alenia Space Inc. (Milano, Italy). As determined by SR-microCT, compared with the mice individually housed in control ventilated cages, the MIS mice revealed no significant changes in either 3D microarchitecture or mineralization degree in any of the investigated bone sites (calvaria, spine, and femur).

Three missions have provided the first documented data on in-flight mice skeletal changes taking advantage of the use of high-resolution X-ray microCT: the 12-day shuttle mission (STS-108) with 2-month-old C57BL6/J female mice, the 15-day shuttle mission (STS-131) with 16-week-old female C57BL/6J mice, and the 91-day mission aboard the ISS with 2-month-old wild-type C57BLJ10 (WT) and pleiotrophin-transgenic (PTN-Tg) male mice.

In the first experiment, Lloyd (Lloyd et al., 2015) tested the ability of Osteoprotegerin-Fc (OPG-Fc) to preserve bone mass during the spaceflight (SF). Twelve mice per group were injected, 24 h prior to launch, with OPG-Fc or an inert vehicle (VEH). Ground control (GC) mice (VEH and OPG-Fc) were kept in environmental conditions mimicking those in the space shuttle, while the age-matched baseline (BL) controls were sacrificed before the launch. MicroCT (μCT20; Scanco Medical AG; Brüttisellen, Switzerland) was used to investigate the trabecular bone architecture with the following experimental parameters: pixel size of 9 μm, scan settings of 55 KVp, 145 mA, and 200 ms per projection (pp). The scanning sites were the trabecular portion immediately distal to the growth plate in the tibiae and humerus. The trabecular bone parameters included: trabecular bone volume fraction (BV/TV); connectivity density (Conn.D); trabecular number (Tb. N); trabecular separation (Tb.Sp); and (SMI). In the tibia, the BV/TV of SF/VEH was 26% lower than GC/VEH, while the Conn.D was 27% lower (although not significantly), the Tb.Th was 16% lower, and the SMI was 6% greater. The spaceflight did not produce modification on the same parameters when SF/OPG-Fc mice were compared to GC/OPG-Fc. Both BV/TV and Conn.D were not changed by spaceflight in the humerus site. In synthesis, this microCT study showed that a single treatment with OPG-Fc before the flight efficiently prevented the detrimental effects of microgravity on mouse bone.

In the second experiment, Blaber (Blaber et al., 2013) exposed eight mice to microgravity to test if osteocytic osteolysis, and cell cycle stopping during osteogenesis may contribute to bone resorption in microgravity conditions. MicroCT (SkyScan 1174 microCT scanner, Kontich, Belgium) was used to image and quantify bone morphometry of the ischium region in the right coxa. Images were acquired with the following experimental parameters: pixel size of 6.77 μm, scan settings of 50 KV, 800 mA, and 3.5 s per projection (pp). These analysis of the pelvis showed that microgravity induced a decrease in BV/TV of 6.29%, and in bone thickness (B.Th) of 11.91%, without reducing the bone mineral density (BMD).

**Figure 1:**
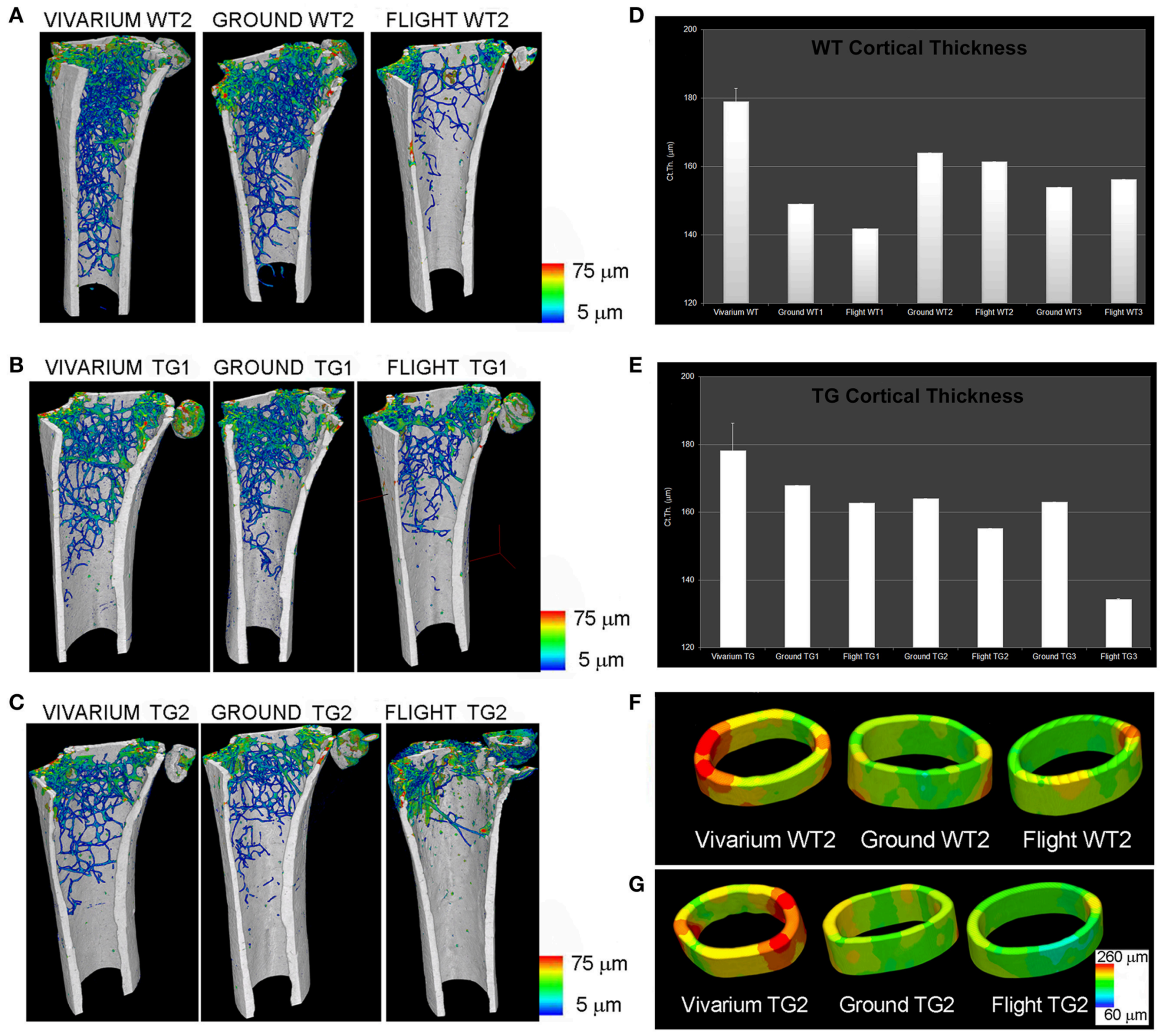
MDS spaceflight mission. Femurs of mice housed for 3 months in the International Space Station (ISS). **(A–C)** Wt2 **(A)**, PTN-Tg1 **(B)**, PTN-Tg2 **(C)** color map of trabecular thickness distribution of vivarium (representative sample), ground and flight mice. **(D,E)** Quantification of cortical thickness distribution in Wt **(D)** and PTN-Tg **(E)** vivarium control (average on 3 mice), ground and flight mice. **(F,G)** Cortical thickness color maps of representative 3D reconstructions in Wt2 vivarium, ground and flight **(F)** and in PTN-Tg2 vivarium, ground, and flight **(G)**. **(A–C)** were originally published in Figure 1 of (Tavella et al., 2012). **(D–G)** were originally published in Figure 3 of (Tavella et al., 2012).

Afterwards, during the Italian Mice Drawer System (MDS) mission, six mice were exposed for 91 days to microgravity on the ISS (Cancedda et al., 2012). This spaceflight is, to date, the longest one ever experimented: for this reason, it has provided a broad range of results, including insights of the PTN transgene possible protection against bone loss due to microgravity (Tavella et al., 2012). SR-microCT imaging was performed at the SYRMEP Beamline of the ELETTRA Synchrotron Radiation Facility (Trieste, Italy), using a beam energy of 19 keV over 180° and with a resulting pixel size of 9 μm. Investigations were focused for the weight-bearing sites on the lower third of the left femurs from the patella toward the shaft of the femur (in the trabecular and cortical portions), and onto the vertebral body in the VII lumbar ring. In non-weight-bearing bones, the analysis was restricted to the middle of the parietal bone left portion (transverse direction from the sagittal suture to the border). SR-microCT analysis revealed a bone loss during spaceflight in the weight-bearing bones of both WT and PTN strains, with a decrease of the trabecular number (Tb.Nr) as well as an increase of the trabecular separation (Tb.Sp) after flight (Figure 1). Non-weight-bearing bones were shown to be not affected by microgravity constrains.

The Bion-M1 mission offered another opportunity to characterize, by microCT, the skeletal changes in adult (23-weeks-old) male C57/BL6 mice after 30-day spaceflight and an 8-day recovery period (Gerbaix et al., 2017). Like the MDS mission, two ground control groups were included in the protocol: a Habitat Control group, which was kept in the same spacecraft cages; and a Control group, which was kept in standard cages. All left femurs, L3, and T12 vertebrae (5/6 animals per group) were scanned with a high-resolution microCT device (VivaCT40, Scanco Medical, Bassersdorf, Switzerland), using a pixel size of 12.5 μm and an analysis protocol previously adopted and described by (David et al., 2003). Comparing the ground control groups, the spacecraft cage confinement was found to negatively affect the femur and lumbar vertebrae (but not the thoracic vertebrae): indeed, the L3 vertebrae and femur trabecular BV/TV and Conn.D were decreased in the Habitat Control vs. the Control group. Moreover, the trabecular BV/TV of the L3 vertebrae decreased in the Flight group vs. both the Habitat Control and Control groups (−35.7 and −56.5%, respectively; *p* < 0.0033): this was attributed to decreased Tb.N and Tb.Th. Flight group femurs showed a large loss of trabecular BV/TV when compared to both the Control and Habitat Control groups (−85.2%, *p* < 0.0003; −64.8%, *p* < 0.017; respectively). Similar trabecular parameters were found in the Flight + Rec and Flight groups, indicating that 1 week of restored gravity is not sufficient to start bone structure recovery.

In the same study (Gerbaix et al., 2017), five cortical femur sections per group were also investigated by SR-microCT, at the ID19 beamline of the European Synchrotron Radiation Facility (Grenoble, France), with a 0.7 μm pixel size, 2000 projections over a total angle of 360° and 26 keV beam energy. The 3D analysis was focused on the several thousands of osteocyte lacunae, getting data on total lacunar volume (Lc.V, mm^3^), lacunar number density (N.Lc/TV) and lacunar volume density Lc.V/TV (%). Being the shape of an osteocyte lacunae approximate to an ellipsoid, the authors used the second-order moments to efficiently measure the lengths of the main axis of the best fitting ellipsoid. The canal volume fraction (Ca.V/TV, %) was also determined. This sophisticated analysis showed that, in the Flight animal group, the osteocyte lacunae had smaller volume with more spherical shape. Furthermore, the number of empty lacunae were significantly (+344%) increased respect to the Habitat Control group. These data demonstrated that microgravity caused osteocyte death, possibly responsible of bone resorption with the consequent bone mass loss.

The 30-day Bion-M1 mission also offered the chance to study six male C57BL/6 mice (19–20 weeks old). The animals were sacrificed 13–15 h after landing. Eight control mice were kept on the ground during the same 30-day period in standard vivarium cages. Caudal motion segments from flight and vivarium mice were loaded to failure in four-point bending (Berg-Johansen et al., 2016). After this test, the same motion segments were imaged by microCT (μCT 50, SCANCO Medical, Brüttisellen, Switzerland) to quantify trabecular microarchitecture and BMD. Motion segments scanned using a 4 μm pixel size and the following scanning parameters: 55 kVp and 109 μA.

It was observed that spaceflight significantly reduced vertebral BV/TV, BMD, and Tb.Th., possibly explaining the tendency of flight specimens to fail within the epiphyseal bone. These microCT results, combined with the mechanical bending tests data, indicate that vertebral bone loss during spaceflight may compromise spine bending capacity, contributing to increased disc herniation risk in astronauts.

However, despite the novelty of the results of the two missions previously described (the 91-day MDS and the 30-day Bion-M1), their experimental protocols appeared deficient in the management of the control mice that were located on the ground instead of in space. Indeed, this choice may have affected the results, preventing reliable conclusions on the impact of microgravity on rodents. More specifically, locating control mice on the ground instead of in space disregarded some factors, like cosmic radiation, microbial environment, flight vibration, shock, and semi-steady acceleration during the launch and return phases inside the space vehicle.

Thus, Shiba (Shiba et al., 2017) developed a novel experimental platform to generate artificial gravity in space. This study was conducted in the framework of a Japan Aerospace Exploration Agency (JAXA) project focused on elucidating the impacts of partial gravity (*partial g*) and microgravity (μ*g*) on mice using newly developed mouse habitat cage units (HCU) that were implemented in the Centrifuge-equipped Biological Experiment Facility in the ISS. In the first mission of the project, 12 five-week-old C57BL/6 J male mice were housed under μg or artificial earth-gravity (*1 g*). μg mice floated inside the HCU, whereas artificial *1 g* mice were on their feet (on the bottom floor of the HCU, at a centrifugation 77 rpm). After 35 days, all mice were returned to the Earth and investigated. The right femurs of mice were analyzed by microCT, using a ScanXmate-A100S Scanner (Comscantechno,Yokohama, Japan). MicroCT revealed that artificial gravity loading significantly reduced bone loss induced by microgravity conditions. Significant decreases were evident in femur bone density of μ*g* mice, whereas artificial *1 g* mice maintained the same bone density as mice in the ground control experiment, in which housing conditions in the flight experiment were replicated. These data further confirmed that the previous experiments' bone loss was specifically due to changes in gravity.

## Conclusions and perspectives

Most of the data from these mice studies were in agreement with those previously obtained from human studies, confirming the utility of the mouse model in the spaceflight investigations. Despite this, the results should be analyzed with caution and attention, considering the specific experimental conditions, such as animal age, sex, body weight, pregnancy, and other variables, possibly influencing the study output (Tavella et al., 2012).

Indeed, as summarized in Table 1, the materials (mouse strain, age, sex, bone site, etc.) and methods (flight duration, animal management, microCT resolution and settings, etc.) reported in literature differed also considerably among themselves. This fact constitutes an incentive to continue research on animal models submitted to spaceflight experience, on the one hand standardizing the survey parameters, on the other by deepening the studies of bone superstructure by nanotomography, possibly based on synchrotron radiation (Langer et al., 2012; Pacureanu et al., 2012; Hesse et al., 2015), as already explored by Maude (Gerbaix et al., 2017) after the 30-day Bion-M1 mission.

**Table 1:** Main spaceflight mission experiences: parameters and results on the mice skeletal bone architecture studied by high-resolution tomography (microCT).

**Project**	**Mice**	**Investigated Bone Sites**	**X-Ray MicroCT Parameters**	**Main Results**
STS-108 (12-day)	2-month-old C57BL6/J (female)	Tibias, humerus	μCT20; Scanco Medical AG; Brüttisellen, Switzerland pixel size: 9 μm, scan settings: 55 kVp, 145 mA	Humerus microarchitecture not changed by spaceflight; Single OPG-Fc treatments before the flight prevented the detrimental effects of microgravity on mouse bone^*^
STS-131 (15-day)	16-week-old C57BL/6J (female)	Ischium region in the right coxa	Scanner 1,174; SkyScan; Kontich, Belgium pixel size: 6.77 μm, scan settings: 50 kV, 800 mA	Microgravity induced ischium bone loss without reducing BMD^**^
MDS (91-day)	2-month-old C57BLJ10 and pleiotrophin-transgenic (male)	Femur, spine, calvaria	SYRMEP beamline; ELETTRA Synchrotron Facility; Trieste, Italy pixel size: 9 μm, beam energy: 19 keV	Microgravity induced bone loss in weight-bearing sites (femur and spine); Parietal bone microarchitecture apparently not changed by spaceflight; PTN transgene possible protection against bone loss^§^
Bion-M1 (30-day)	23-week-old C57/BL6 (male)	Left femurs, L3 and T12 vertebrae	VivaCT40, Scanco Medical, Bassersdorf, Switzerland Pixel size:12.5 μm, scan settings: n.a.	Microgravity negatively affected the femur and lumbar vertebrae, but not the thoracic vertebrae; Bone microstructure recovery was not initiated with one week of recovery training^#^
		Cortical femur	ID19 beamline; ESRF Synchrotron Facility; Grenoble, France pixel size: 0.7 μm, beam energy: 26 keV	Spaceflight induces osteocyte death, possibly responsible for bone resorption and consequent bone loss^#^
	19/20-weeks-old C57BL/6 (male)	Caudal motion segments of the spine	μCT 50, SCANCO Medical, Brüttisellen, Switzerland pixel size: 4 μm, scan settings: 55 kVp, 109 μA	Microgravity significantly reduced vertebral bone volume fraction, BMD, and trabecular thickness; Vertebral bone loss during spaceflight may degrade spine bending properties^##^
JAXA (35-day)	5-week-old C57BL/6 J (male)	Right femurs	ScanXmate-A100S Scanner; Comscantechno, Yokohama, Japan pixel size: n.a., scan settings: n.a.	Artificial *g* loading significantly suppressed bone loss induced by microgravity conditions^+^

* Lloyd et al., 2015;

** Blaber et al., 2013;

§ Tavella et al., 2012;

# Gerbaix et al., 2017;

## Berg-Johansen et al., 2016;

+ *Shiba et al., 2017*.

Moreover, the possibility to simulate an artificial loading on the musculoskeletal system, as in hypergravity conditions (Canciani et al., 2015), represents an undoubted advantageous tool for scientific research, since it may allow researchers to avoid the complexity of real microgravity studies and makes possible the investigation of a larger number of subjects, thus improving the power of such studies.

## Author contributions

AG: Concept and design; revision of the whole literature; coordination of the work drafting; final version definition and approval. SM: Design (high-resolution tomography); data research in literature on microCT; work drafting; final version approval. AR and BC: Design (physiology concepts); data research in literature on physiological interpretation of data; work drafting; final version approval. RC and ST: Concept and design; data research in literature on spaceflight Projects; work drafting; final version definition and approval. All the authors agreed to be accountable for all aspects of the work in ensuring that questions related to the accuracy or integrity of any part of the work are appropriately investigated and resolved.

### Conflict of interest statement

The authors declare that the research was conducted in the absence of any commercial or financial relationships that could be construed as a potential conflict of interest.
